# Hemobilia and Melena After Liver Biopsy: A Case Report and Review of Literature

**DOI:** 10.4021/gr569w

**Published:** 2013-10-31

**Authors:** Mohamed H Emara, Ibrahim M Ibrahim, Mohamed I Radwan, Mohamed RAbd Elbast

**Affiliations:** a Tropical Medicine Department, Faculty of Medicine, Zagazig University, Zagazig, Egypt; b Department of Radiology, Refaat Scan Center, Biala, Egypt

**Keywords:** Hemobilia, Melena, Liver biopsy, Gall bladder hemtoma.

## Abstract

Liver biopsy is the gold standard for assessment of hepatic fibrosis although it is associated with many complications. We reported a 28-year-old chronic HCV patient who developed gall bladder hematoma with hemobilia and melena after liver biopsy. The hematoma resolved with conservative management.

## Introduction

Formation and accumulation of fibrosis in the liver is the common pathway that leads to progressive liver disease. Precise staging of liver fibrosis is essential for patient management in clinical practice because the presence of bridging fibrosis represents a strong indication for antiviral therapy. Liver biopsy (LB) has always been represented as the standard of reference for assessment of this hepatic fibrosis, although it has several limitations [1].

## Case Report

**Figure 1:**
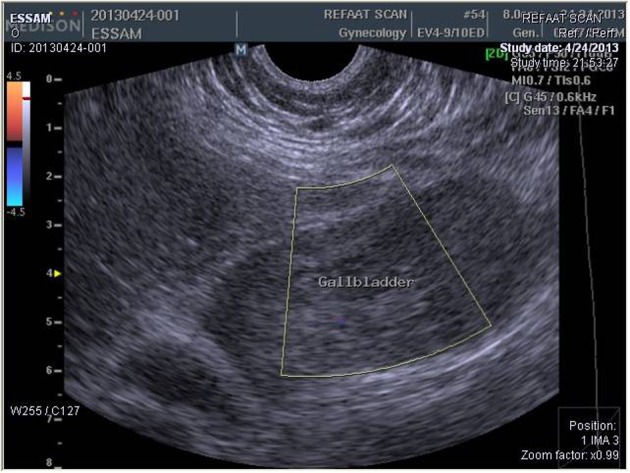
Abdominal ultrasound with color flow doppler showing huge gall bladder hematoma filling the whole lumen with absence of color flow waves.

**Figure 2:**
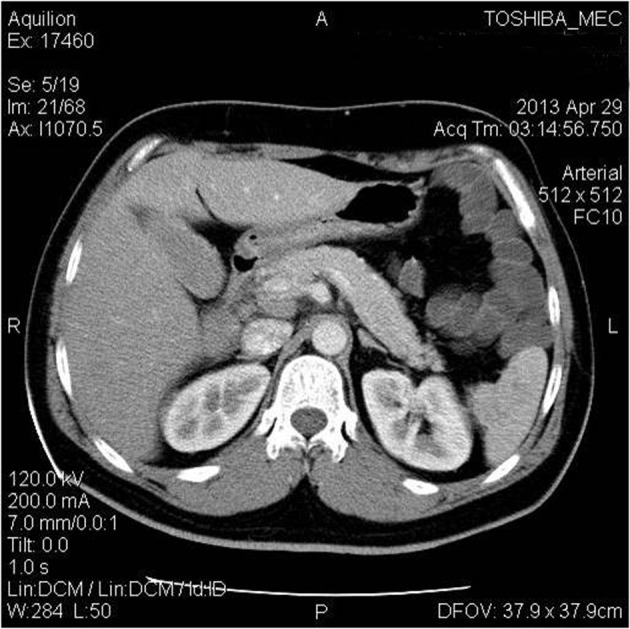
Abdominal computed tomography showing resolution of the gall bladder hematoma.

A 28-year-old male patient with chronic HCV underwent a percutaneous liver biopsy for enrollment in the interferon based therapy according to the national guidelines. Three days after liver biopsy the patient experienced severe colicky right hypochondrial pain associated with low grade fever, vomiting and melena. In the next day he noticed dark coloration of urine and a tinge of jaundice in his eyes. His data are shown in Table 1. He thought medical advice, where we examined him and he had blood pressure of 100/70 mmHg, pulse was regular with 82 beats/min, and he was jaundiced. Abdominal ultrasound with color flow doppler examination showed large hematoma filling the gall bladder lumen (Fig. 1), this was not found in the pre-biopsy ultrasound report (done one week prior to liver biopsy). A clinical suspicion of post liver biopsy hemobilia due to gall bladder injury and hematoma was proposed. The patient was examined one day later by upper endoscopy that was free. His lab data after the liver biopsy are shown in Table 1. The patient was candidate for conservative management at an outpatient basis with antibiotics and analgesics and was instructed for emergency hospital admission if he experienced increasing pain, bleeding manifestations or any other major events. Daily telephone communication for follow-up and case monitoring was done. One week later (about 12 days after liver biopsy) an abdominal triphasic computed tomography scans were done (Fig. 2) and showed resolution of the heamtoma. The patient was maintained on general tonics to achieve a target hemoglobin > 12 g/dL till he began to receive pegylated interferon therapy.

**Table 1:** The Patient's Characteristics

Items	Value
Baseline data	
Body mass index	25
Prothrmobin activity	76%
ALT (IU/L)	117(42)
AST (IU/L)	150 (42)
Hemoglobin (g/dL)	13.6
Platelets /mm^3^ × 10^3^	195
Total bilirubin (mg/dL)	0.4
After liver biopsy	
Hepatic activity (METAVIR)	A2
Hepatic fibrosis (METAVIR)	F3
Hemoglobin (g/dL)	11.4
Total bilirubin (mg/dL)	7

## Discussion

Liver biopsy is an invasive procedure that is widely used to diagnose and assess different liver diseases including chronic HCV, but it is difficult to be accepted by many patients; even many patients may discourage antiviral therapy due to their fear from LB [2]. It is associated with many complications; although the rate of major complications is around 2-4% and that is why it is performed as an outpatient procedure [3].

Hemorrhagic complications and perforations are the most devastating complications of liver biopsy. Bleeding is usually presented as subcapsular or parenchymal hematoma, free intraperitoneal hemorrhage, hemobilia or rarely hemothorax. Hemorrhage will be recognized within 4 hours after biopsy. Delayed bleeding has been reported as late as 15 days after biopsy [4].

It has been reported in two large studies of patients post liver biopsy that bleeding occur in 0.32-0.35% with morbidity related to hemorrhage of 0.24% and around 0.11% had mortality from severe bleeding [5, 6].

The least common of the hemorrhagic complications of liver biopsy is hemobilia. Hemobilia is a term first used in 1946 by Sandblom to describe bleeding into the bile ducts [7]. A review of hemobilia cases by Sandblom indicated that trauma was the cause of hemobilia in half of the cases, with one third of these hemobilia cases being iatrogenic and needle biopsy of the liver represented the most common cause [8]. Hemobilia is not common after liver biopsy, where in the study of Piccinino et al [5], 4 patients with hemobilia out of 86,276 liver biopsies were reported with a rate of 0.00005%.

The bleeding is usually arterial in origin and can be venous in patients with portal hypertension. Biopsy can induce hematoma or pseudoaneurysm into bile duct and delayed bleeding can occur from gradual dissolution of that clot, while acute bleeding which is less common is secondary to simultaneous perforation of intrahepatic bile ducts and blood vessels [9].

Gall bladder injuries after LB are rare with only a handful of cases reported in the literature. One case of gall balder injury and associated biliary peritonitis was reported in a 30-year-old chronic HBV patient [3], while an extreme case of intra-peritoneal hemorrhage following percuatenous LB in patient with chronic pancreatitis extended to the scrotum was also reported [10].

Hemobilia is usually suspected when a post procedure fall in hemoglobin is associated with the classic triad of abdominal pain, hyperbilirubinemia, and unexplained gastrointestinal bleeding with average onset of approximately five days after biopsy [5, 9].

Clinical presentation ranges from chronic anemia to rapid massive bleeding with hematemsis and/or melena and rarely patient may present with only hematochezia [11].

Diagnosis is usually suspected in patients with history of recent liver biopsy. Radiological modalities can help in diagnosis. Ultrasonography with color flow Doppler can identify a needle track sign [12], gall bladder and hepatic hematomas, leakage and intra-abdominal free fluid. Computed tomography and magnetic resonance imaging are also highly valuable. However, the best of radiological modalities is selective angiography, because it can accurately identify the source of bleeding and offer a therapeutic approach at the same setting. Endoscopic examination may reveal the blood oozing from the duodenal papilla [11].

Hemobilia usually stops spontaneously or with intravenous fluids and blood products [13], and if patient still have continuous or intermittent bleeding then immediate selective angiography is needed as diagnostic and therapeutic tool and considered to be the cornerstone of management [11]. Surgical exploration for major bleeding is indicated if hemodynamic instability persists despite the above measures and the laparotomy rate amongst patients who bled range from 6% to 25% [13].

Gall bladder injury after LB was controlled by conservative management with iv fluids, antibiotics and analgesics in many cases [3, 10]. However, cholecystectomy may be needed [14].

The case presented here is important due to many reasons. Firstly, it documents a major complication; bleeding of this frequently performed outpatient procedure. Secondly, it report gall bladder injury; a rare complication of LB, this may raise a suspicion about less experience of the operator and consequently favors ultrasound guided approach while performing LB [3]. Also the needle system used in this case (Tru-cut) is known to be associated with more complication when compared with the modified Minghini needles [3], the rate of complications is expected to be higher when used for cases with advanced fibrosis (F3 in our case). Fourthly, diagnostic work-up should begin whenever complications anticipated and expectation of major complication should never be overlooked. Fifthly, conservative management is always successful in these patients but armamentarium for emergencies must be an integral part of patient management. Lastly, centers performing LB should raise both operator's and patient's awareness about complications of LB and should implement strategies for dealing with complications including post-procedural monitoring of patients for a time, guarantee communication upon development of any adverse events and facilitate emergency service for patients who already developed complaints.
